# Comparative Study of the Potential Cell-Penetrating Peptide ∆M4 on Apoptosis Cell Signaling in A375 and A431 Cancer Cell Lines

**DOI:** 10.3390/pharmaceutics16060775

**Published:** 2024-06-07

**Authors:** Estefanía Fandiño-Devia, Aleksandra Brankiewicz, Gloria A. Santa-González, Ibeth Guevara-Lora, Marcela Manrique-Moreno

**Affiliations:** 1 Group of Structural Biochemistry of Macromolecules, Faculty of Exact and Natural Sciences, University of Antioquia, A.A. 1226, Medellin 050010, Colombia; liliana.fandino@udea.edu.co; 2 Department of Analytical Biochemistry, Faculty of Biochemistry, Biophysics and Biotechnology, Jagiellonian University, 30-387 Krakow, Poland; aleksandra.brankiewicz@doctoral.uj.edu.pl; 3 Doctoral School of Exact and Natural Sciences, Jagiellonian University, 30-387 Krakow, Poland; 4 Grupo de Investigación e Innovación Biomédica, Facultad de Ciencias Exactas y Aplicadas, Instituto Tecnológico Metropolitano, A.A. 54959, Medellin 050010, Colombia; gloriasanta@itm.edu.co

**Keywords:** anticancer peptides, cell-penetrating peptides, melanoma, epidermoid carcinoma, apoptosis, oxidative stress

## Abstract

In recent yearsjajajj, peptide-based therapeutics have attracted increasing interest as a potential approach to cancer treatment. Peptides are characterized by high specificity and low cytotoxicity, but they cannot be considered universal drugs for all types of cancer. Of the numerous anticancer-reported peptides, both natural and synthetic, only a few have reached clinical applications. However, in most cases, the mechanism behind the anticancer activity of the peptide is not fully understood. For this reason, in this work, we investigated the effect of the novel peptide ∆M4, which has documented anticancer activity, on two human skin cancer cell lines. A novel approach to studying the potential induction of apoptosis by anticancer peptides is the use of protein microarrays. The results of the apoptosis protein study demonstrated that both cell types, skin malignant melanoma (A375) and epidermoid carcinoma (A431), exhibited markers associated with apoptosis and cellular response to oxidative stress. Additionally, ∆M4 induced concentration- and time-dependent moderate ROS production, triggering a defensive response from the cells, which showed decreased activation of cytoplasmic superoxide dismutase. However, the studied cells exhibited a differential response in catalase activity, with A375 cells showing greater resistance to the peptide action, possibly mediated by the Nrf2 pathway. Nevertheless, both cell types showed moderate activity of caspases 3/7, suggesting that they may undergo partial apoptosis, although another pathway of programmed death cannot be excluded. Extended analysis of the mechanisms of action of anticancer peptides may help determine their effectiveness in overcoming chemoresistance in cancerous cells.

## 1. Introduction

Antimicrobial peptides (AMPs) are molecules known for their unique ability to extensively interact with the cell membranes of microorganisms. The most accepted mechanism of action is based on their amphipathic nature and positive charge, which allows the peptide to bind to and disrupt the integrity of bacterial cell membranes, leading to increased permeability and, ultimately, cell death [1,2,3]. However, it has been demonstrated that peptides also employ different pathways to enter the cell; these molecules are known as cell-penetrating peptides (CPPs) [4]. CPPs are short peptides of up to 40 amino acids with the ability to gain access to the cytoplasm by different mechanisms, including endocytosis, to promote an intracellular effect [5,6]. Given the similarities between the membranes of cancer and microbial cells, there is growing interest in exploring the potential of AMPs, now referred to as anticancer peptides (ACPs), as novel therapeutic agents for cancer treatment. In particular, cationic peptides have demonstrated promising selective cytotoxicity against cancer cells due to their preferential interaction with negatively charged membranes. Currently, significant efforts are being made to screen and evaluate over 3000 ACPs for their anticancer activity [7].

In contrast to normal cells, cancer cells exhibit distinct characteristics such as hypoxia, elevated levels of reactive oxygen species (ROS), and alterations in lipid and carbohydrate metabolism, resulting in changes in the composition of their cell membranes. These changes include overexpression of phosphatidylserine (PS), increased levels of zwitterionic phosphatidylethanolamine, and abnormal glycosylation patterns of glycolipids and glycoproteins [8]. Furthermore, the loss of membrane asymmetry in cancer cells can lead to reduced membrane permeability, posing challenges for drug delivery [9]. On the other hand, these features of cancer cells predispose them to interact with cationic peptides, leading to the destruction of the cell membrane by mechanisms similar to those observed with microorganisms, ultimately leading to cell death [7]. In addition to the direct peptide interaction with cell membranes, ACPs have been found to modulate various intracellular processes within cancer cells. Several preclinical studies have demonstrated the wide potential of natural and synthetically modified peptides as effective anticancer agents [10,11,12,13,14,15,16]. The proposed mechanisms of peptide action include the induction of apoptosis, promotion of necrotic cell death, inhibition of DNA replication, suppression of angiogenesis, and modulation of immunity mechanism [17,18].

ΔM4 is a synthetic derivative of cecropin D that has demonstrated promising anticancer activity in human skin cell lines [15,19]. This study aimed to investigate the potential mechanisms underlying the effects of this peptide on two human skin cancer cell lines, A375 and A431. This is important because the effectiveness of CPPs depends, in part, on the number of PS in the outer layer of the cell membrane, and it has been documented that these cells differ significantly in this respect. The A375 cells have almost twice the PS content compared to the A431 cells [20]. Our research compares the changes induced by this peptide in regulating oxidative stress and intracellular signaling associated with the control of programmed cell death in both cell lines.

## 2. Materials and Methods

### 2.1. Cell Cultures

A375 (melanoma, ATCC CRL-1619, purchased from ATCC https://www.atcc.org/products/crl-1619, accessed on 10 April 2024) and A431 (epidermoid carcinoma, CRL-1555, purchased from ATCC https://www.atcc.org/products/crl-1555, accessed on 10 April 2024) skin cell lines were cultured in Dulbecco’s modified Eagle’s medium supplemented with 5% fetal bovine serum, 100 µg/mL penicillin, and 100 µg/mL streptomycin. The cells were seeded and cultured to achieve 80–100% confluence at 37 °C in a humidified incubator with 5% CO_2_. Before the experiments, cells were examined under a microscope to verify the correct morphology, adherence, and exponential growth. All reported data included at least three independent experiments per treatment group.

### 2.2. Determination of Apoptosis Protein Expression

Cell cultured in 75 cm^2^ bottles were treated with 12.5 µM ∆M4 (NFFKRIRRAWKRIWKWIYSA) (GenScript, Piscataway Township, NJ, USA) for 24 h. After treatment, cells were rinsed with PBS, and the proteins were isolated for immunodetection using the Human Apoptosis Array Kit (R&D Systems, Minneapolis, MN, USA) following the manufacturer’s instructions. The supernatants were subjected to protein quantification using the BCA test (Thermo Fisher Scientific, Waltham, MA, USA). Equal amounts of proteins (400 μg) were used for apoptosis analysis. The intensity score of each duplicated array spot was measured with the Gel Doc XR+ Gel Documentation system with Image Lab™ 6.1.0.07 Software (Bio-Rad, Hercules, CA, USA). The membranes were exposed to the ray film for 5 min, with 60 exposure times per minute. The average background signal was subtracted, and the protein expression was presented as pixel density. The identity and respective coordinates of all the antibodies on the arrays can be found in the “Supplementary Materials” (Table S1).

### 2.3. ROS Production Assay

For the assessment of intercellular ROS production, an NBT assay was performed according to Brankiewicz et al. [21]. Briefly, cells (1.5 × 10^4^) were seeded on a 96-well plate and stimulated with peptide at concentrations 2.5, 5.0, and 12.5 µM for different periods. After treatment, cells were incubated with 0.8 mg/mL NBT (BioShop, Burlington, ON, Canada) solution in RPMI 1640 medium w/o Phenol Red. The absorbance of the dissolved crystals formed was measured with Synergy 463H1 microplate reader (Agilent, Santa Clara, CA, USA) at 620 nm. The results were normalized to the values obtained for control cells at the appropriate incubation time, which was assumed to be 100%.

### 2.4. Determination of Antioxidant Activity

To assess catalase (CAT) and superoxide dismutase (SOD) activity, cells (8 × 10^5^) were seeded on Petri cell culture dishes (ϕ100 mm) and incubated with peptide in 5.0 and 12.5 μM concentrations. Incubation was carried out for 3, 6, and 12 h. Subsequently, the cells were scraped and lysed in 50 mM Tris buffer supplemented with 3 mM MgSO_4_, 100 mM Tricine, 3 mM EGTA, 1 mM DTT, and pH 8.0. Enzyme activity was measured according to published protocols [22]. The activity of two SOD isoforms: MnSOD and Cu/ZnSOD, was analyzed by densitometry using Quantity One 4.6.8 software (BioRad, Hercules, CA, USA). The results were presented as a relative change in comparison to untreated cells assumed to be 100%.

### 2.5. Western Blot Analysis

To evaluate the effect of ΔM4 on apoptotic signaling pathways activation and oxidative stress induction, analysis of p53 and Nrf2 protein levels was performed using the Western blot technique. Cells (2 × 10^5^), seeded on the 12-well plate, were stimulated with 5.0 and 12.5 µM ΔM4. After 6, 12, and 24 h incubation, the proteins were extracted with RIPA buffer (10 mM Tris-HCl, pH 8.0, 1 mM EDTA, 0.5 mM EGTA, 1% Triton X-100, 0.1% sodium deoxycholate, 0.1% SDS, 140 mM NaCl) supplemented with phosphatase and protease inhibitors (1 mM NaVO_4_, 5 mM NaF, 250 μM phenylmethyl sulfonyl fluoride). Equal amounts (5 µg) of proteins were separated by SDS-PAGE electrophoresis in reducing conditions. Proteins were transferred to PVDF membrane (Merck, Darmstadt, HE, Germany) and, after blocking in 5% skim milk, they were developed with primary antibodies (Table S2) overnight at 4 °C. After extensive washing, membranes were then incubated for 1 h at room temperature with secondary HRP-conjugated antibodies (Table S2). Protein bands were visualized with luminol-enhanced substrate Westar Supernova (Cyanagen, Bolonia, E-R, Italy) using ChemiDoc™ XRS+ Imaging System (Bio-Rad, Hercules, CA, USA). Protein expression was analyzed by densitometry using Quantity One 4.6.8 software.

### 2.6. Caspase 3/7 Assay

The activity of caspase-3/7 in cells treated by ΔM4 was evaluated using the Caspase-Glo^®^ 3/7 Assay (Promega, Madison, WI, USA) according to the manufacturer’s instructions. Cells (1 × 10^4^) were seeded in white 96-well plates with clear bottoms and grown for 24 h. Cells were treated with peptide at 5.0 and 12.5 µM concentrations for 6, 12, and 24 h. Luminescence intensity was recorded using Synergy 463H1 microplate reader. Caspase-3/7 activity was expressed as a relative change as compared to non-treated cells.

### 2.7. Statistical Analysis

Statistical analysis of the results for apoptosis array and caspase activity was performed using GraphPad Prism 8.0.1 software (Dotmatics, Bishop’s Stortford, Herts, UK). One-way and two-way ANOVA was performed with post hoc comparisons via Fisher’s Least Significant Difference (LSD) test. Statistics for NBT, catalase activity (One-way ANOVA with Tukey’s post hoc), SOD activity, and WB (one-sample *t*-test) were performed with Statistical 13.3 Software. All data are presented as the mean ± SD. *p*-values below 0.05 were considered statistically significant.

## 3. Results and Discussion

### 3.1. Expression of Apoptotic Proteins by ∆M4 Treatment

In previous studies, we demonstrated significant cytotoxicity of the novel synthetic ∆M4 peptide against skin cancer cells, with a lower cytotoxic effect on a reference non-tumoral HaCaT cell line. The peptide increased membrane permeability in both studied cancer cell lines and caused intensified phosphatidylserine exposure in melanoma cells in concentrations above 25 µM, which suggests the initiation of early apoptosis stages [19]. Furthermore, Santa-Gonzalez et al. [15] proved that ∆M4 caused a change in cell morphology and mitochondrial integrity. In our study, we used a screening test to better understand the promising anticancer effects of this peptide-mediated for a cell-penetrating mechanism, for which we used lower concentrations and shorter exposure times than those reported previously.

**Figure 1:**
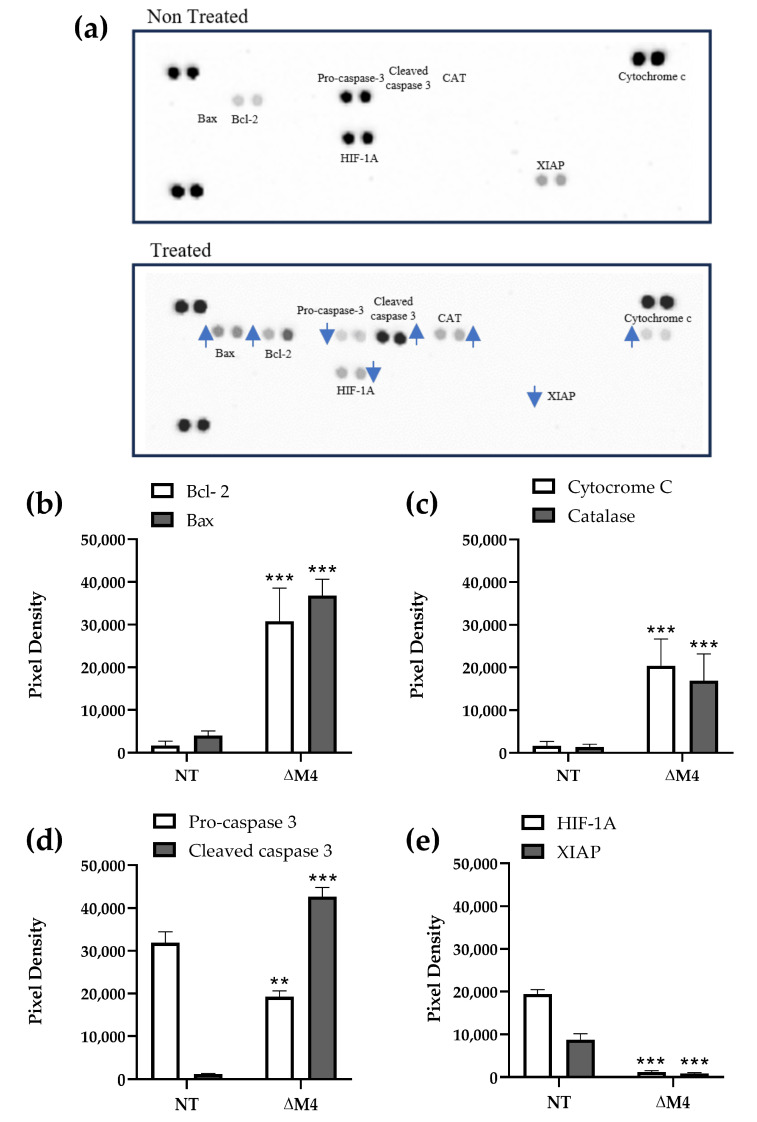
Expression of pro-apoptotic proteins in A375 cells. A representative image of the detection of proteins in non-treated cells (NT) or cells treated with 12.5 μM ∆M peptide for 24 h (**a**). Densitometric quantification of the change in the expression of the proteins: (**b**) Bcl-2 and Bax; (**c**) cytochrome c and catalase; (**d**) pro- and cleaved caspase 3; (**e**) HIF-1A and XIAP. The bars represent the protein expression as the mean pixel ± SD of three independent experiments. Statistical significance of the differences to untreated cells was obtained by one-way ANOVA where ** *p* ≤ 0.01, *** *p* ≤ 0.001. Blue arrows showed an increase (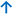) or decrease (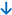) in protein expression.

**Figure 2:**
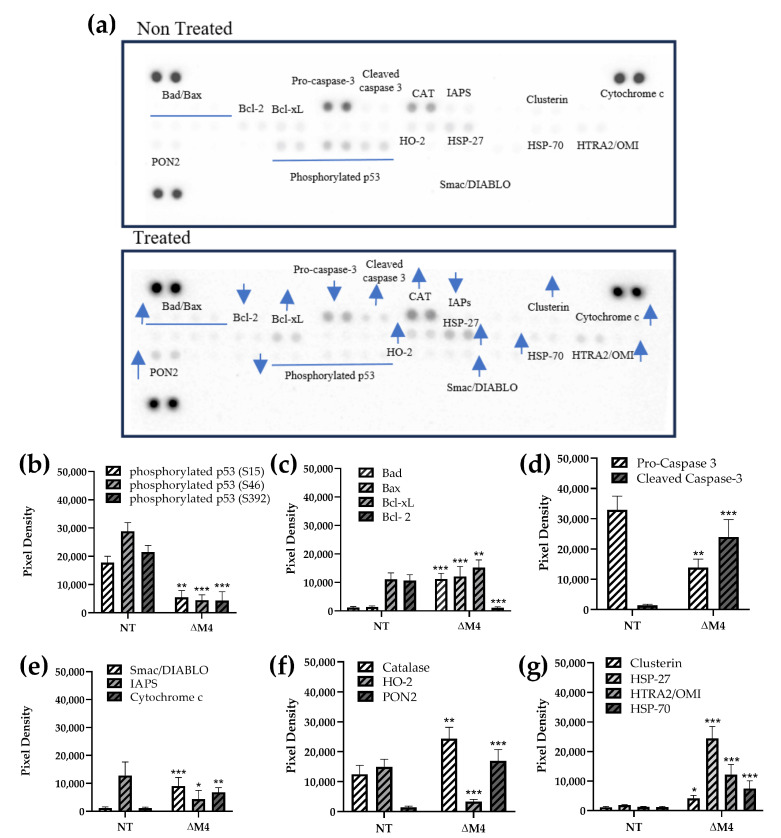
Expression of pro-apoptotic proteins in A431 cells. A representative image of the detection of proteins in non-treated cells (NT) or cells treated with 12.5 μM ∆M peptide for 24 h (**a**). Densitometric quantification of the change in the expression of the proteins: (**b**) phosphorylated p53 proteins; (**c**) Bad, Bax, Bcl-xL, Bcl-2; (**d**) pro- and cleaved caspase 3; (**e**) Smac/DIABLO, IAPS, cytochrome c; (**f**) catalase, HO-2, PON2; and (**g**) clusterin, HSP-27, HtrA2/Omi, HSP-70. The bars represent the protein expression as the mean pixel density ± SD of three independent experiments. Statistical significance of the differences to non-treated cells was obtained by one-way ANOVA where * *p* ≤ 0.05, ** *p* ≤ 0.01, *** *p* ≤ 0.001. Blue arrows showed an increase (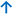) or decrease (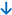) in protein expression.

The microarray assay was performed to assess changes in the expression of 35 proteins involved in apoptosis signaling. These results indicated that ∆M4 caused both up- and down-regulation in at least 8 proteins of 35 apoptotic proteins tested in the array (the expression increased or decreased by up to 30-fold compared to untreated cells) (Figure 1 and Figure 2). In the A375 cell line, the peptide significantly induced upregulation of two proteins involved in intrinsic apoptosis B-cell lymphoma-extra-large (Bcl-2) and Bcl-2-like protein 4 (Bax) (Figure 1b). Bcl-2 is considered an apoptosis inhibitor, while Bax is a pro-apoptotic protein. Although these results seem to contradict the concept of a pro-apoptotic effect of the peptide, it should be mentioned that anti-apoptotic proteins can be blocked by interaction with the p53 protein [23]. 

Moreover, the expression of catalase and cytochrome c was also increased, indicating a raised cellular stress response [24] (Figure 1c). The decisive signal indicating the activation of apoptosis pathways was an increased level of cleaved caspase-3, the enzyme responsible for protein degradation during apoptosis (Figure 1d). This effect was accompanied by a decreased level of procaspase-3. Furthermore, the decreasing expression of the X-linked inhibitor of apoptosis protein (XIAP), a caspase inhibitor [25], confirms the above findings. We also observed a significant decrease in the level of the hypoxia-inducible factor 1 (HIF-1A), a transcription factor leading to the overexpression of several growth factors (Figure 1e) that regulate vascularization, angiogenesis, energy metabolism, and cell survival [26]. Therefore, the obtained results lead to the conclusion that A375 cells enter apoptosis under the influence of ∆M4.

Microarray screening results revealed more significant differences in the A431 cell line after ∆M4 treatment (Figure 2a). The levels of various forms of phosphorylated p53 protein were significantly reduced (Figure 2b). The p53 protein is a tumor suppressor that controls cell proliferation and apoptosis [27]. Our results suggest that reduced p53 activation may contribute to the enhancement of pro-apoptotic processes. In contrast to A375 cells, we observed a different profile of the regulatory proteins of apoptosis after peptide treatment in A431 cells (Figure 2c). The expression of anti-apoptotic Bcl-2 decreased, while the level of pro-apoptotic proteins Bax and Bad increased. Notably, Bcl-2 and Bcl-xL bind and sequester transmembrane pro-apoptotic proteins, blocking cytochrome c release from mitochondria, in turn leading to cell survival [24].

Our screening proved that ΔM4 was able to alter the expression of these proteins, promoting cell apoptosis. Indeed, similar to A375 cells, in A431, we also observed a significant increase in active cleaved caspase 3 and a decrease in procaspase 3 (Figure 2d). These changes indicate an intrinsic apoptosis pathway [25], which was supported by the elevated release of the secondary mitochondrial activator of caspases (Smac/DIABLO) and the decreased level of apoptosis inhibitors (IAPs) (Figure 2e). Similar to A375 cells, in A431 cells, the peptide also induced high cytochrome c release (Figure 2e), indicating a loss of the mitochondria membrane integrity.

After ∆M4 treatment, A431 cells also exhibited altered levels of cellular stress-related proteins, such as catalase (CAT), heme oxygenase 2 (HO-2), and transmembrane protein paraoxonase 2 (PON2) (Figure 2f). A significant increase in catalase level induced in both cell lines treated with ΔM4 indicates an antioxidant response of the cells. Similarly, an increase in PON2 suggests an enhanced anti-stress response. PON2 has been associated with oxidative stress control, inhibition of extrinsic apoptosis, prevention of superoxide formation, and increased release of cytochrome c, as well as the progression of various types of malignancies [28,29]. In turn, HO-2, a constitutive enzyme involved in cell homeostasis, is assumed to be responsible for regulating the inflammatory and reparative cell response to injury [30]. In this study, we observed a decreased expression of this enzyme, suggesting an opposite effect after ΔM4 administration. Elevated levels of the serine protease Htr2/Omi, involved in mitochondrial homeostasis, also suggest a pro-apoptotic action of ∆M4 (Figure 2g). Intriguingly, we also observed an increased expression of heat shock proteins (HSPs) such as clusterin, HSP-27, and HSP-70, which inhibit apoptosis and promote survival in cancer cells [31] (Figure 2g).

In general, our screening study showed that ∆M4 has evident pro-apoptotic potential in the tested cell lines, although some mechanisms indicating cellular defense against the action of this peptide may occur, especially in the A431 cell line. Regarding the different PS content in the outer membrane of these cells compared to A375, it appears that the peptide–membrane interaction can differ and possibly depend on the peptide concentration and incubation time. Therefore, further studies were undertaken to investigate the impact of the peptide concentration and incubation time on the oxidative stress levels in the examined cells to clarify whether different mechanisms may contribute to cell death.

### 3.2. ∆M4 Increases Intracellular ROS Production

**Figure 3:**
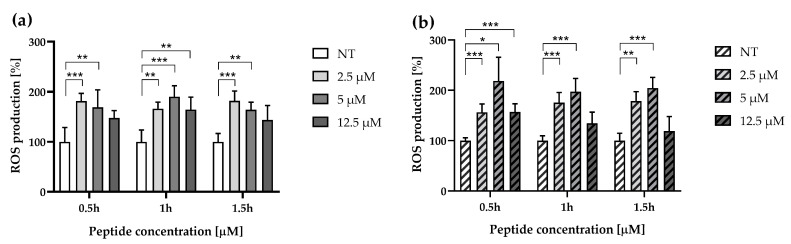
Effect of ΔM4 on intracellular ROS generation. ROS production in A375 (**a**) and A431 (**b**) cells was measured after the treatment with 2.5, 5.0, and 12.5 μM of ΔM4 for 0.5, 1.0, and 1.5 h. Bars represent mean ± SD of values compared to the control (basal value = 100%). Differences to untreated cells were obtained by two-way ANOVA with Tukey’s post hoc test, where * *p* ≤ 0.05, ** *p* ≤ 0.01 and *** *p* ≤ 0.001.

To investigate the impact of ∆M4 on cellular oxidative stress, we initially assessed the ROS level at different peptide concentrations (up to 12.5 μM) and at different incubation times (up to 1.5 h) (Figure 3). The results demonstrated a significant increase in ROS production in both cell lines. Lower peptide concentrations (up to 5 μM) result in more effective ROS production, especially with short incubation times. The peptide concentration of 12.5 μM caused significant changes after 1 h in A375 cells and after 0.5 h in the second cell line. Overall, at this concentration, the peptide induces lower ROS production regardless of the incubation time compared to untreated cells.

In cancer cells, the balance of ROS is crucial for cell signaling pathways leading to either cell survival or death. Moderate increases in ROS levels can activate cell defense pathways that promote proliferation, migration, protection, and drug resistance in cancerous cells [32]. Conversely, excessively high intracellular ROS levels can lead to regulated cell death when the cell defenses are insufficient. A recent study revealed the strong pro-apoptotic action of brevilaterin B, a peptide derived from *Brevibacillus laterosporus*, which acted selectively against A431 cells with increased ROS production [33]. Moreover, our previous study showed that ∆M4 induces oxidative stress in skin cancer cells, leading to mitochondrial membrane damage, which may enhance apoptotic processes [15]. Due to the identification of markers of defensive processes in the examined cells under the influence of the ∆M4 peptide, further research was undertaken to determine how these cells are defended against oxidative stress.

### 3.3. ∆M4 Lowers the Cu/ZnSOD Activity in Melanoma and Epidermoid Carcinoma Cells

**Figure 4:**
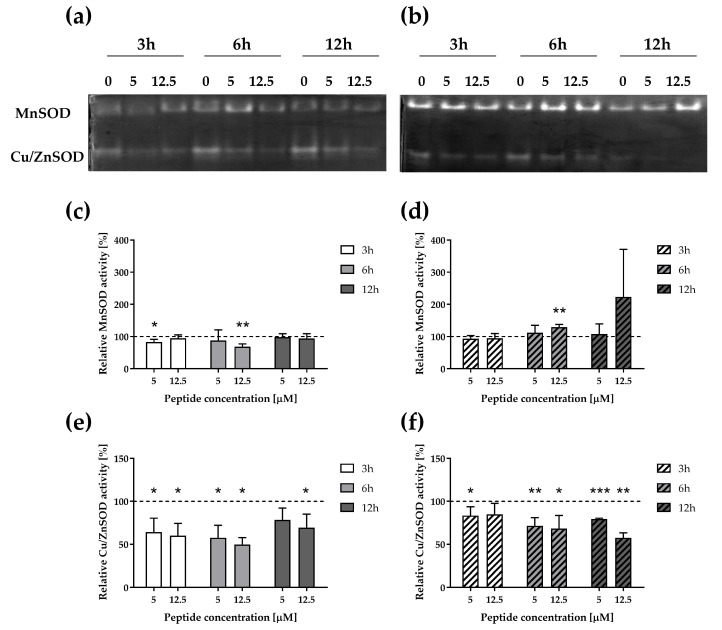
Impact of ΔM4 on SOD activity in A375 and A431 cell lines. Cells were treated for 3, 6, and 12 h with ΔM4 at different concentrations. The activity of SOD isoforms for A375 cells (**a**) and A431 cells (**b**) was visualized on native PAGE gels stained with NBT. After densitometric quantification, MnSOD and Cu/ZnSOD activities were presented for A375 (**c**,**e**) and A431 (**d**,**f**) cell lines. Bars represent mean ± SD compared to the control values of untreated cells, assumed to be 100% (dashed lines). Differences to untreated cells were obtained by one sample *t*-test, where * *p* ≤ 0.05, ** *p* ≤ 0.01, and *** *p* ≤ 0.001.

The appearance of a stressor in the cellular environment triggers the activation of defense mechanisms. The main natural source of ROS is the mitochondrial electron transport chain, which generates superoxide anion, which in turn is dismutated to H_2_O_2_ by SOD. Subsequently, H_2_O_2_ is decomposed to oxygen and water by catalase [34]. To assess the response of cells to the peptide-induced oxidative stress, the activity of two SOD isoforms was analyzed: cytoplasmatic Cu/ZnSOD (SOD1) and the mitochondrial MnSOD (SOD2) (Figure 4a). Our results showed decreased Cu/ZnSOD activity in both studied cell lines treated by ∆M4, with minor changes in MnSOD activity (Figure 4). It appears that the decrease in the activity of this enzyme in A375 cells occurs more rapidly than in the second cell line, where the greatest decrease was observed only after 12 h.

The results of Cu/ZnSOD activity were surprising because we expected increased activity of SOD, one of the first-line defense enzymes, particularly in A431 cells, which exhibited increased expression of many markers associated with protection against oxidative stress. It can be assumed that the dynamics of peptide penetration into both types of cells may differ due to the different lipid composition of their membranes, and therefore, A431 cells can scavenge the produced ROS but at a later stage, allowing the cells to activate their defense systems. Additionally, previous studies have demonstrated that inhibiting these enzymes can induce apoptosis in cancer cells [35,36,37], which may confirm the pro-apoptotic effect observed in this study (Figure 1 and Figure 2). Cu/ZnSOD is localized in the intermembrane space of the mitochondria, where it protects them from the irreversible oxidative damage caused by superoxide [38]. Therefore, it can be assumed that the loss of Cu/ZnSOD activity may explain the pro-apoptotic effect of the tested peptide in the examined cells.

### 3.4. ∆M4 Decreases Catalase Activity in A375 Whereas It Has No Significant Effect in A431

**Figure 5:**
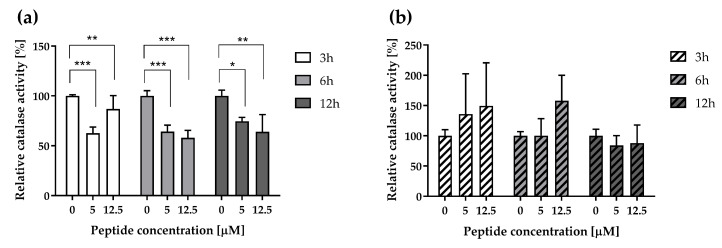
Impact of ΔM4 on catalase activity. Cell lines A375 (**a**) and A431 (**b**) were treated with ΔM4 for 3, 6, and 12 h. Bars represent mean ± SD of values compared to the control (basal value = 100%). One-way ANOVA with Tukey’s post hoc test was performed. * *p* ≤ 0.05, ** *p* ≤ 0.01 and *** *p* ≤ 0.001.

Screening results revealed an increase in catalase protein level following 24 h incubation of cells with the peptide (Figure 1 and Figure 2). For this reason, the activity of catalase, a mitochondrial antioxidant enzyme involved in ROS scavenging [33], was assessed. Surprisingly, in contrast to the elevated catalase expression after 24 h peptide treatment, the activity of this enzyme decreased significantly in A375 cells, and this effect was found to be time- and peptide concentration-dependent (Figure 5a). Interesting and contrasting results were observed in A431 cells, wherein there was a slight tendency for increased catalase activity at shorter treatment durations (Figure 5b). Therefore, the peptide treatment elicited distinct cellular responses among the tested cell lines. 

As summarized by Glorieux et al. [39], cancer cells typically exhibit reduced catalase activity, which correlates with elevated intracellular H_2_O_2_ levels, thereby activating signaling pathways associated with proliferation, migration, and invasion. These authors also reported that the expression of catalase in cancer cells is upregulated due to gene promoter activation by various transcription factors, although the specific mechanisms remain to be fully elucidated. It is plausible that ∆M4 activates similar pathways in the examined cells, leading to increased catalase protein levels in both cell lines; however, these cells may employ different mechanisms to counteract oxidative stress. Indeed, it has been shown that under physiological conditions, the activity of catalase is increased by the mediating antioxidant functions of p53, which protect the cell against endogenous ROS, while after toxic stress, high levels of p53 and p53-inducible gene 3 interact to inhibit catalase activity, which leads to a shift in the oxidant/antioxidant balance towards oxidative status, promoting apoptotic cell death [40].

In addition, earlier studies have suggested that membrane-associated active catalase can protect cells against the ROS-dependent apoptotic pathway, potentially contributing to cellular resistance [41,42]. As can be observed, there is no change in catalase activity after a prolonged incubation time with the peptide in A431 cells, while in the second cell line, the activity of this enzyme continues to decrease. This difference could also be attributed to the dynamic of the peptide penetration. Thus, the peptide-induced expression of various anti-apoptotic factors in A431 cells may be explained by the dual action of catalase, whereby some cells can develop resistance while others undergo apoptosis. 

### 3.5. ∆M4 Change Expression of the Stress–Response Transcription Factors in A375 and A431 Cell Lines

**Figure 6:**
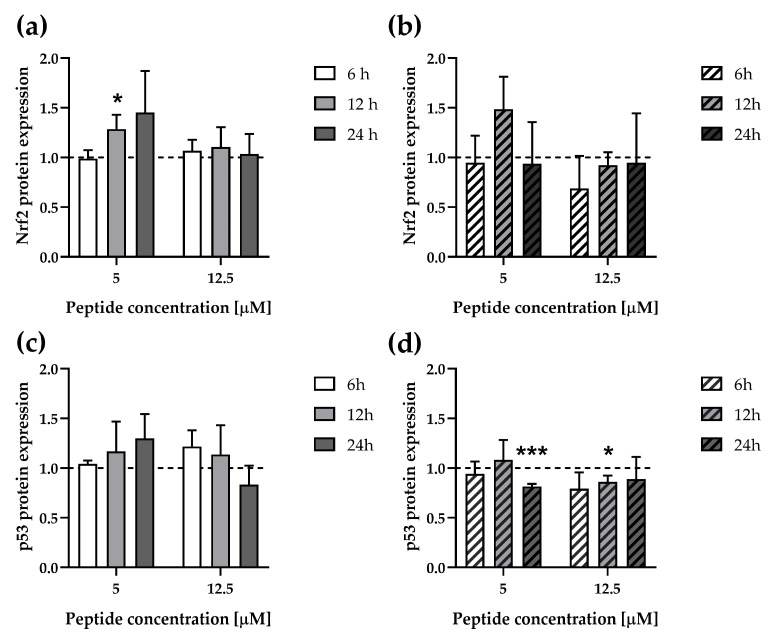
Impact of ΔM4 on selected protein expressions in A375 and A431 cell lines. Expression of Nrf2 in A375 and A431 cells ((**a**,**b**), respectively). Expression of p53 in A375 and A431 cells ((**c**,**d**), respectively). Cells were treated for 6, 12, and 24 h with 5 and 12.5 μM of ΔM4. Bars represent mean ± SD values calculated by densitometry from Western blot images. The dashed lines represent the value of untreated cells normalized to the value of 1.0. Differences to untreated cells were obtained by one sample *t*-test, where: * *p* ≤ 0.05 and *** *p* ≤ 0.001.

The aforementioned results prompted us to investigate key cellular stress–response pathways. One key stress response factor is the nuclear factor erythroid-derived-2-like 2 (Nrf2), which is responsible for activating the antioxidative response element sequence (ARE). Nrf2 binding to ARE is controlled by Kelch-like ECH-associated protein 1 (Keap1), which mediates Nrf2 ubiquitination and its subsequent proteasomal degradation [43]. Although Nrf2 primarily regulates oxidative cell responses by inducing genes involved in various metabolic pathways, including glutathione metabolism, ROS and xenobiotics detoxification, NADPH regeneration, and iron and heme metabolism [44], it has also been associated with a dual role in cancer development. Nrf2 hyperactivation may promote cancer development by protecting against apoptosis processes, thereby promoting cell growth and resistance to chemo- and radiotherapy. Our observations revealed different effects of ∆M4 on the tested cells. A375 cells exhibited time-dependent increased expression of Nrf2 at low peptide concentrations, whereas higher peptide concentrations resulted in basal protein expression (Figure 6a). These results may suggest that A375 cells, with increased Nrf2 content, may exhibit an enhanced anti-stress response, especially since Nrf2 regulates CAT expression [32]. However, the decreased Nrf2 expression observed at higher peptide concentrations, which may lead to enhanced cell penetration, can be related to the altered anti-stress response. In contrast, Nrf2 expression in A431 appeared to be down-regulated only at high concentration after a 6 h incubation (Figure 6b), correlating with the findings from the screening study (Figure 2), where upregulated expression of anti-apoptotic proteins was observed.

Another key player in stress-dependent pathway activation is the p53 protein, which primarily functions to maintain genomic stability and integrity. In response to stress, p53 undergoes post-translational modifications and promotes the transcription of genes involved in various processes, including cell cycle arrest, apoptosis, autophagy, ferroptosis, cell metabolism, and ROS generation [23]. Our study revealed concentration-dependent changes in p53 expression in A375 cells, with increasing trend of expression observed at low peptide concentration over time, whereas higher concentration resulted in decreased expression, suggesting that higher peptide concentrations may promote apoptosis due to compromised cellular defense mechanisms (Figure 6c). This interpretation correlates with the increased Nrf2 expression in the results discussed above. Conversely, p53 expression in treated A431 cells remained relatively low compared to untreated cells (Figure 6d). It is worth noting that these results pertain to the entire p53 protein pool, while screening assays showed a significant decrease in p53 phosphorylation under the influence of the peptide. The absence of phosphorylation prevents the formation of p53 tetramers, hindering its binding to DNA and inhibiting the transcriptional activity of its target gene [23,45,46]. It is worth underlining that A431 cells express a mutant form of the *TP53* gene with substitution in the codon 273 (His273) related to loss in function at the transcription level [47]. The A431 cell line expresses only 50% of the normal level of p53 mRNA because only one allele was present, and the second is mutated. In addition, the p53 phosphorylation at S15 and S46 is important for stress response, and its loss can lead to defects in cell cycle checkpoints and DNA repair. Therefore, our results could suggest that ΔM4 may inhibit the function of p53 and influence its response to stress conditions, potentially through alternative apoptotic pathways. For example, p53 executes signals not only directly as a transcription factor but also from its interaction with other proteins, such as anti- and pro-apoptotic proteins in the mitochondria [23].

### 3.6. ∆M4 Induces Activation of Effector Caspases 3/7

**Figure 7:**
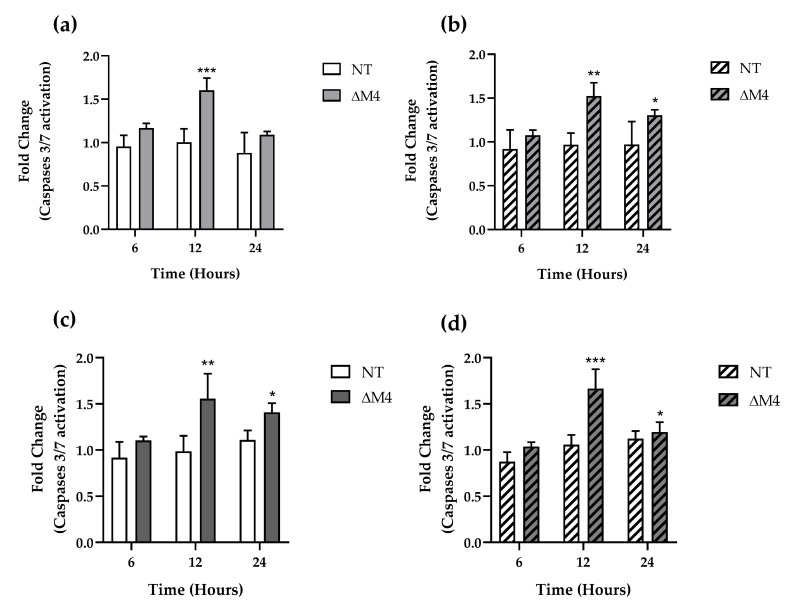
Activation of caspases 3/7 in cells under the influence of ΔM4. Cells A375 (**a**,**c**) and A431 (**b**,**d**) were treated with ΔM4 a 5 µM (**a**,**b**) and 12.5 µM (**c**,**d**) for 6, 12, and 24 h. The bars show the enzymatic activity of caspases 3/7 expressed as the mean ± SD of three independent experiments as compared to the values obtained for non-treated cells, which were normalized to a value of 1. The differences in non-treated cells were obtained by one-way ANOVA. * *p* ≤ 0.05, ** *p* ≤ 0.01, and *** *p* ≤ 0.001.

Finally, to determine whether the cell death-inducing effect of ΔM4 is associated with apoptosis, the activation of the effector caspases 3/7 was assessed. During apoptotic cell death, the extrinsic or intrinsic activation pathways converge through initiator caspases 8 and 9, respectively, to activate effector caspases 3 and 7 [48]. Different concentrations and incubation times with the peptide were employed to better understand how caspases 3/7 are activated under the influence of ΔM4. Significant changes in the activation of caspases 3/7 were observed in both cell lines following treatment with ΔM4 (Figure 7). 

Both cell lines exhibited a statistically significant increase in caspase activation after incubation with low peptide concentration (5 μM) (Figure 7a,b). The most effective incubation time was 12 h, with significant activation of caspases after 24 h, albeit to a lesser extent. Additionally, a higher concentration of ΔM4 resulted in increased activation of caspase 3/7 in both cell types, not only after 12 h but also after 24 h (Figure 7c,d). These observations are consistent with the results obtained from microarray screening, where the level of cleaved caspase 3 was also higher in treated cells. It is important to note that these concentrations are below the IC_50_ for these cells. At low concentrations, peptides can transiently open the cell membrane, leading to its destabilization [49]. The caspases 3/7 results suggest that the peptide ΔM4 may penetrate the cell membrane and induce apoptosis through an intrinsic pathway. Indeed, increased cytochrome c levels were observed in these cells after peptide treatment, which correlates with previous findings from our working group that demonstrated ΔM4-induced changes in mitochondrial membrane potential and increased ROS concentration in A375 and A431 cells, providing evidence for apoptosis induction.

## 4. Conclusions

Our research demonstrated the pro-apoptotic effects in the A375 melanoma cell line and the A431 epidermoid carcinoma cell line induced by the potential cell-penetrating peptide ΔM4. The oxidative stress may likely be involved in the development of apoptosis; however, another type of regulated cell death cannot be excluded. It seems that the studied cell lines can undergo apoptosis by different pathways that may be related to the concentration of the peptide and the dynamics of its penetration. Such processes are dependent on the composition of lipids on the outer cell membrane. The use of peptides in anticancer therapies is constantly developing. Therefore, elucidating the mechanisms of new candidate peptides that are responsible for cell death in various types of cancer cells may contribute to obtaining additional information necessary for further development of such therapeutics. Nevertheless, the use of more sophisticated biophysical techniques, such as Transmission Electron Microscopy or Atomic Force Microscopy, will help to elucidate the potential internalization of ΔM4 in the cells. Moreover, intensive studies in in vivo models can contribute to determining the anticancer potential of the peptide ΔM4.

## Data Availability

The data involved in this paper are presented in articles and supporting materials in the form of diagrams or tables.

## Supplementary Materials

The following supporting information can be downloaded at: https://www.mdpi.com/article/10.3390/pharmaceutics16060775/s1, Table S1: Location coordinates of the apoptotic protein array; Table S2: A list of antibodies used in this research.
