# Supplementary material for: Comparative Study of the Potential Cell-Penetrating Peptide ∆M4 on Apoptosis Cell Signaling in A375 and A431 Cancer Cell Lines
Source: Pharmaceutics. 2024 Jun 7;16(6):775. doi: 10.3390/pharmaceutics16060775 (PMC11207241; doi:10.3390/pharmaceutics16060775)
Supplement: Supplementary file 1 [file pharmaceutics-16-00775-s001.zip › pharmaceutics-3021017-supplementary.pdf]

Supplementary materials

Table S1. Location coordinates of the apoptotic protein array

Coordinate	Target/Control
A1, A2	Reference Spots
A23, A24	Reference Spots
B1, B2	Bad
B3, B4	Bax
B5, B6	Bcl-2
B7, B8	Bcl-x
B9, B10	Pro-Caspase-3
B11, B12	Cleaved Caspase-3
B13, B14	Catalase
B15, B16	cIAP-1
B17, B18	cIAP-2
B19, B20	Claspin
B21, B22	Clusterin
B23, B24	Cytochrome c
C1, C2	TRAIL-R1/DR4
C3, C4	TRAIL-R2/DR5
C5, C6	FADD
C7, C8	Fas/TNFRSF6/CD95
C9, C10	HIF-1α
C11, C12	HO-1/HMOX1/HSP32
C13, C14	HO-2/HMOX2
C15, C16	HSP27
C17, C18	HSP60
C19, C20	HSP70
C21, C22	HtrA2/Omi
C23, C24	Livin
D1, D2	PON2
D3, D4	p21/CIP1/CDKN1A
D5, D6	P27/Kip1/ CDKN1B
D7, D8	Phosphorylated p53 (S15)
D9, D10	Phosphorylated p53 (S46)
D11, D12	Phosphorylated p53 (S392)
D13, D14	Phosphorylated Rad17 (S365)
D15, D16	Smac/DIABLO
D17, D18	Survivin
D19, D20	TNFR1/TNFRSF1A
D21, D22	XIAP
D23, D24	PBS (negative control)
E1, E2	Reference Spots

Table S2. A list of antibodies used in this research**.**

Protein	Primary antibody	Dilution	Specification	Secondaryantibody	Dilution	Specification
β-actin	mouse	1:10 000	3800S, Cell Signalling Technology	anti-mouse, conjugated with HRP	1:10 000	Cell Signalling Technology
p53	mouse	1:2000	sc-126, Santa Cruz Biotechnology		1:4000	
Nrf2	rabbit	1:2000	16396-1-AP, Proteintech	anti-rabbit, conjugated with HRP	1:5000	Cell Signalling Technology
